# Treatment outcome according to genetic tumour alterations and clinical characteristics in digestive high-grade neuroendocrine neoplasms

**DOI:** 10.1038/s41416-024-02773-w

**Published:** 2024-06-22

**Authors:** Hege Elvebakken, Andreas Venizelos, Aurel Perren, Anne Couvelard, Inger Marie B. Lothe, Geir O. Hjortland, Tor Å. Myklebust, Johanna Svensson, Herish Garresori, Christian Kersten, Eva Hofsli, Sönke Detlefsen, Lene W. Vestermark, Stian Knappskog, Halfdan Sorbye

**Affiliations:** 1 Department of Clinical and Molecular Medicine, Faculty of Medicine and Health Sciences, Norwegian University of Science and Technology, Trondheim, Norway; 2 Department of Oncology, Ålesund Hospital, Møre and Romsdal Hospital Trust, Ålesund, Norway; 3 K.G. Jebsen Center for Genome-Directed Cancer Therapy, Department of Clinical Science, University of Bergen, Bergen, Norway; 4 Institute of Tissue Medicine and Pathology, University of Bern, Bern, Switzerland; 5 Department of Pathology, Université Paris Cité and AP-HP, Bichat Hospital, Paris, France; 6 Department of Pathology, Oslo University Hospital, Oslo, Norway; 7 Department of Oncology, Oslo University Hospital, Oslo, Norway; 8 Department of Research and Innovation, Møre and Romsdal Hospital Trust, Ålesund, Norway; 9 Department of Registration, Cancer Registry Norway, Oslo, Norway; 10 Department of Oncology, Sahlgrenska University Hospital, Gothenburg, Sweden; 11 Department of Oncology, Stavanger University Hospital, Stavanger, Norway; 12 Department of Research, Hospital of Southern Norway, Kristiansand, Norway; 13 Department of Oncology, St.Olavs Hospital, Trondheim, Norway; 14 Department of Pathology, Odense University Hospital, Odense, Denmark; 15 Department of Clinical Research, Faculty of Health Sciences, University of Southern Denmark, Odense, Denmark; 16 Danish Medicines Agency, Copenhagen, Denmark; 17 Department of Oncology, Haukeland University Hospital, Bergen, Norway; 18 Department of Clinical Science, University of Bergen, Bergen, Norway

**Keywords:** Cancer genetics, Gastrointestinal cancer

## Abstract

**Background:**

Chemotherapy has limited efficacy in advanced digestive high-grade neuroendocrine neoplasms (HG-NEN) and prognosis is dismal. Predictive markers for palliative chemotherapy are lacking, and prognostic markers are limited.

**Methods:**

Digestive HG-NEN patients (*n* = 229) were prospectively included 2013–2017. Pathological re-assessment revealed 188 neuroendocrine carcinomas (NEC) and 41 neuroendocrine tumours (NET G3). Tumour-DNA was sequenced across 360 cancer-related genes, assessing mutations (mut) and copy number alterations. We linked sequencing results to clinical information and explored potential markers for first-line chemotherapy efficacy and survival.

**Results:**

In NEC given cis/carboplatin and etoposide (PE), *TP53*mut predicted inferior response rate in multivariate analyses (*p* = 0.009) and no BRAFmut NEC showed response. In overall assessment of PE-treated NEC, no genetic alterations were prognostic for OS. For small-cell NEC, *TP53*mut were associated with longer OS (*p* = 0.011) and *RB1* deletions predicted lack of immediate-progression (*p* = 0.003). In non-small cell NEC, *APC* mut were associated with immediate-progression and shorter PFS (*p* = 0.008/*p* = 0.004). For NET G3, *ATRX*mut, *ARID1A*- and *ERS1* deletions were associated with shorter PFS.

**Conclusion:**

Correlations between genetic alterations and response/immediate-progression to PE were frequent in NEC but affected PFS or OS only when subdividing for cell-type. The classification of digestive NEC into large- and small-cell seems therefore molecularly and clinically relevant.

## Introduction

Neuroendocrine neoplasms (NEN) encompass a variety of neoplasms with different phenotypes, ranging from indolent low-grade neuroendocrine tumours (NET) to aggressive neuroendocrine carcinoma (NEC). The 2019 WHO classification of digestive high-grade NEN (HG-NEN) separated well-differentiated NET G3 from poorly differentiated NEC, both with a high proliferation defined by Ki-67 > 20% [1]. Digestive NET G3 and NEC entities differ in clinical and molecular characteristics, response to treatment and prognosis [2–5]. Metastatic NEC is associated with an especially poor prognosis, with median overall survival ranging from 1 month if untreated, to 11–12 months if given chemotherapy [6–8]. Established first-line palliative chemotherapy consisting of cis/carboplatin and etoposide is extrapolated from treatment of small-cell lung cancer (SCLC) based on clinical and morphological similarities [9–11]. The treatment has been unchanged since the 1990s, as has the dismal prognosis. Up to 30% of digestive NEC have an immediate disease progression evident at first radiological evaluation after receiving cis/carboplatin and etoposide. Progression free survival (PFS) is only 4–5 months. Metastatic NET G3 have a better prognosis compared to NEC, with a median overall survival (OS) of 33–36 months, although the efficacy of cis/carboplatin and etoposide has been reported inferior [4, 5, 12].

The implementation of next generation sequencing has led to a changed paradigm of cancer treatment by identifying genetic alterations targetable for treatments, with great success across a variety of cancers. Such molecular aberrations have also shown to be of value as predictors of treatment effect [13]. In a recent comprehensive molecular overview assessing digestive HG-NEN with a targeted approach covering 360 genes, we reported differences in both mutational frequency and genes affected when comparing NEC and NET G3 and a clear difference compared to previous reports on SCLC. In the same study, we found potentially targetable aberrations in 66% of 152 included NEC [3]. In a whole genome sequencing approach examining 16 NEC, 94% were reported to have potential targetable molecular alterations [14]. To date, few studies have assessed the clinical impact of molecular alterations in NEC [15, 16]. Thus, reliable biomarkers to guide treatment choice and to predict efficacy of therapy and survival for digestive HG-NEN are lacking. The aim of the present study was to evaluate the impact of genetic alterations on treatment outcomes and survival in a large cohort of digestive HG-NEN. In addition, through identification of potential molecular drivers among digestive HG-NEN, we aimed to elucidate potential targets eligible for future targeted treatment approaches.

## Methods

### Patients

Patients diagnosed with HG-NEN with digestive primary, or unknown primary with a predominance of gastrointestinal metastases were included prospectively from 2013–2017 from nine Scandinavian secondary and tertiary hospitals. Clinical data were available through the Nordic NEC Registry. Patients (*N* = 180) from our recent study on molecular characteristics of digestive HG-NEN were included. These patients were selected based on available tumour and matched normal tissue, allowing for the analysis of somatic copy number alterations (CNA) [3]. In addition, 49 novel digestive HG-NEN cases without matched normal tissue were included. As such, 229 patients were included in the present analyses. Formalin-Fixed-Paraffin-Embedded tumour tissue and tumour sections (haematoxylin/eosin, synaptophysin, chromogranin A and Ki-67) were collected for all patients. Patients were enroled prior to the formal introduction of NET G3 among digestive HG-NEN [1]. To ensure correct stratification according to the 2019 WHO classification, all cases were blinded and re-evaluated digitally in 2021-22 by three experienced NEN pathologists (AP, AC and IMBL). Ki-67 was recalculated. Initial ambiguous cases were discussed and decided on in a consensus meeting. Best response was reported according to radiological evaluation by RECIST criteria v 1.1. Mixed Neuroendocrine Non-Neuroendocrine tumours (MiNEN) were excluded (MiNEN covering both small cell NEC/adenocarcinomas and large cell NEC/adenocarcinomas). As the aggressiveness of NEC could prohibit more than one line of treatment, we assessed both immediate progression at first evaluation and response rate (RR) as endpoints assessing treatment efficacy. RR was defined as the proportion of responders (patients with complete or partial response). Immediate progression was defined as the proportion of patients with progression at first evaluation after initiation of first-line chemotherapy. In addition to radiological RECIST progression, three patients with confirmed clinical progression within 2 months of first course of chemotherapy (radiology not performed) were included in the immediate progression group.

### Molecular analyses

Tissue collection, MSI analyses, DNA isolation, library preparation, sequencing, data processing and bioinformatical analyses were performed as described previously [3]. In brief the sequencing analyses covered the coding regions of a targeted panel of 360 cancer related genes. For the 49 cases without normal tissue for filtering of germline variants, somatic mutation calling was restricted to canonical driver mutations, identified following a pre-planned approach as previously described [17]. MSI analyses were available for 180 included patients. To avoid cases with potentially false low TMB, we included only cases with normal tissue for comparison (N = 180).

### Statistics

Categorical variables were presented as percentages and continuous variables as median/means and range, as appropriate. For direct group comparisons, Chi-squared test was applied. The predictive values of categorical variables were explored using logistic regression analyses. PFS was calculated from the start of first-line chemotherapy to the date of progression or last known follow-up. Overall survival was, for the whole unselected cohort (*n* = 229), calculated from the date of diagnosis of high-grade NEN and for those with advanced disease (*n* = 206), from date of metastases/non-resectable disease, both to date of death or last follow-up. Survival curves were estimated using the Kaplan-Meier method, with log rank test for statistical significance. For estimation of hazard ratio, the cox regression model was used. Variables with a *p* < 0.10 in univariate analyses or assumed of particular potential clinical value (the majority pre-defined) were included in multivariate models. As *BRAF* mutated NEC had a zero-response rate to cis/carboplatin and etoposide, this mutation could not be included in the MVA. PS is known as an extremely strong prognostic factor for NEC, and we therefore only analysed cases with PS < 2. *P* values are reported as two-sided and values <0.05 were considered statistically significant. If not otherwise specified, all analyses for NEC were done on the subgroup receiving cis/carboplatin and etoposide (*N* = 123; Table 1), whereas for NET G3, for all chemotherapy regimens combined. The selected genetic alterations in our analyses were predefined as the top 10 alterations. Multiple testing was assessed by Benjamini-Hochberg procedure. Statistical analyses were performed using STATA version 16.1.

**Table 1:** Baseline characteristics for 229 digestive HG-NEN.

	Valid cases	NEN *N* = 229	NET G3 *N* = 41	NEC *N* = 188
Male gender (*n* %)	229	134 (58)	22 (54)	112 (60)
Age in years, median (range)	229	67 (29–90)	65 (38–79)	67 (29–90)
Performance status, *n* (%)^a^	223			
0–1		167 (75)	32 (82)	135 (73)
≥2		56 (25)	7 (18)	49 (27)
Primary tumour site, *n* (%)	229			
Oesophagus		25 (11)	1 (2)	24 (13)
Gastric		20 (9)	2 (5)	18 (9.5)
Pancreas		35 (15)	17 (42)	18 (9.5)
Small bowel		8 (3)	5 (12)	3 (2)
Gallbladder/duct		5 (2)	1 (2)	4 (2)
Colon, right		41 (18)	1 (2)	40 (21)
Colon, left		11 (5)	1 (2)	10 (5)
Rectum		47 (21)	1 (2)	46 (24)
Unknown^b^		33 (14)	11 (27)	22 (12)
Other GI		4 (2)	1 (2)	3 (2)
Ki-67, median (range)	222	80 (21–100)	30 (21–80)	90 (21–100)
Celltype, *n* (%)^a^	186			
Small cell				80 (43)
Non-small cell				106 (57)
Metastases, *n* (%)^a^	229			
Synchronous		187 (82)	36 (88)	151 (80)
Metachronous		14 (7)	2 (5)	12 (6)
Resection of primary tumour, *n* (%)^a^	229	70 (31)	12 (29)	58 (31)
First-line palliative chemotherapy, *n* (%)^a^	229	176 (77)	28 (68)	148 (78)
Cisplatin/etoposide		42 (24)	2 (7)	40 (27)
Carboplatin/etoposide		94 (53)	11 (39)	83 (56)
CapTem		19 (11)	10 (36)	9 (6)
Folfirinox/folfoxiri		7 (4)	1 (3.5)	6 (4)
5-FU doublets		4 (2)	0 (0)	4 (3)
Temozolomide		5 (3)	3 (11)	2 (1)
Other medical treatment		5 (3)	1 (3.5)	4 (3)
SRI, *n* (%) Uptake > liver	34	12 (35)	8 (62)	4 (19)
FDG PET uptake, *n* (%)^a^	91	86 (94)	21 (88)	65 (97)
ALP > UNL, *n* (%)^a^	223	122 (55)	24 (60)	98 (54)
LDH > UNL, *n* (%)^a^	209	95 (45)	13 (38)	82 (47)
MSI, *n* (%)^a^	180	9 (5)	0 (0)	9 (6)
TMB, mean (range)	180	4.7 (0–59)	3.2 (0–28)	5.0 (0–59)

To avoid potentially false low TMB only cases where we had normal tissue for comparison (*N* = 180) were included.

*SRI* somatostatin receptor Imaging uptake, mainly octreoscan, *ALP* alkaline phosphatase, *LDH* lactate dehydrogenase, *MSI* microsatellite instable, *TMB* tumour mutation burden, *UNL* upper normal limit.

^a^Presented as fraction of examined patients.

^b^Unknown primary with a predominance of gastrointestinal metastases.

## Results

### Baseline characteristics

After pathological re-evaluation of 229 NEN, 188 (82%) were classified as NEC and 41 (18%) as NET G3. Pancreas was the most common primary tumour site for NET G3, while colorectal primary was most common for NEC. Baseline characteristics are summarised in Table 1. Out of the 229 included patients, 23 underwent curative surgery without evidence of residual disease throughout a median follow-up time of 53.7 months. Of the 207 cases with advanced disease, six had localised non-resectable (NR) disease and 201 had metastatic disease. Further out of these 207, one patient underwent curative surgery, 176 received first-line palliative chemotherapy, 11 radiotherapy, two radio-nucleotide treatment and 17 best supportive care. The majority of NEC were treated with cis/carboplatin and etoposide as first-line palliative chemotherapy (*N* = 123; 80%). For NET G3, 13 (46%) received first-line treatment with cis/carboplatin and etoposide and 10 (36%) with capecitabine/temozolomide.

### Response and survival according to primary NEC site, NEC cell type and differentiation (NET G3 vs NEC)

For NEC given first-line cis/carboplatin and etoposide, RR was 37% whereas 35% had immediate progression at first evaluation after initiation of treatment. RR was 9% among colon NEC (2/22), significantly inferior to a 44% RR for extra-colonic NEC (39/88; *p* = 0.002). RR was better for small-cell NEC (SC-NEC) compared to large cell NEC (LC-NEC): 48 vs 26% *p* = 0.019. We found no significant difference in RR to cis/carboplatin and etoposide comparing NET G3 (*N* = 12) and NEC (*N* = 110; 37 vs 42%, *p* = 0.766).

We found no difference in PFS depending on primary NEC site, comparing colon to extra-colonic (2.1 vs 4.1 m, *p* = 0.170) or pancreatic to non-pancreatic (3.3 vs 5.6 m, *p* = 0.076). For patients given cis/carboplatin and etoposide there were no difference in PFS comparing SC- and LC-NEC (4.9 vs 2.4 m, *p* = 0.076), nor comparing NET G3 and NEC (3.6 vs 3.3 m, *p* = 0.577).

Assessing the whole cohort, we found a significant longer OS for NET G3 compared to NEC (22.2 vs 8 m, *p* < 0.001). The difference between NET G3 and NEC was significant for those given chemotherapy in general (18.6 vs 8.5 m, *p* < 0.001) but not for those given cis/carboplatin and etoposide (11.1 vs 8.8 m, *p* = 0.143). We found no difference in OS after cis/ carboplatin and etoposide when comparing SC and LC-NEC (9.2 vs 8.5 m, *p* = 0.53), nor when comparing colon to extra-colonic NEC (8.4 vs 8.9 m, *p* = 0.743) or pancreatic to non- pancreatic NEC (11.1 vs 8.7 m, *p* = 0.151).

### Response and survival according to clinical biomarkers

For NEC patients given cis/carboplatin and etoposide, performance status (PS) did not influence RR, but PFS was longer with PS 0–1 compared to PS ≥ 2 (4.1 vs 2.2 m, *p* = 0.004), as were OS (10.2 vs 4.7 m, *p* < 0.001). In a univariate model, significantly inferior OS for NEC was seen for CRP > 10 (7.4 vs 11 m, *p* = 0.047) and elevated lactate dehydrogenase (LDH) (6.3 vs 11.1 m, *p* = 0.032), but without affecting RR or PFS.

### Genetic alterations and treatment outcome in NEC

**Fig. 1:**
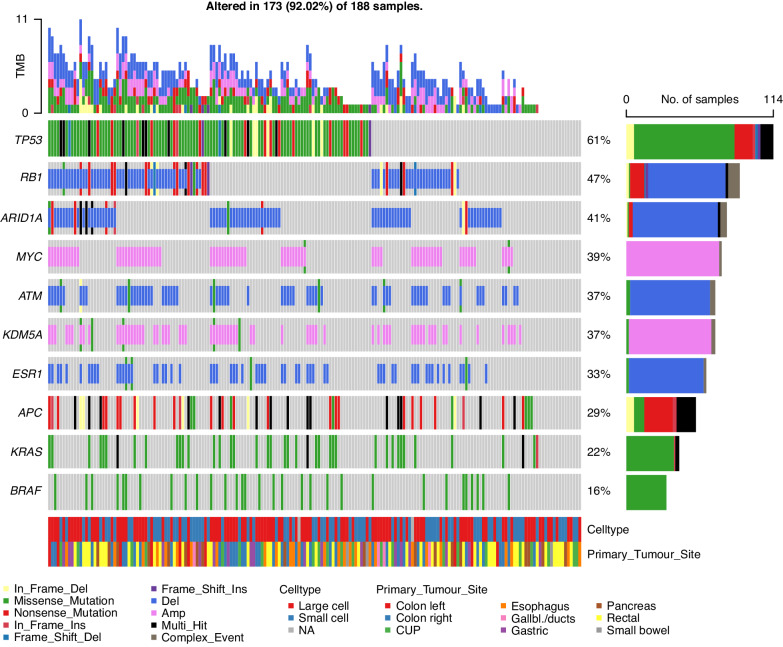
Top genetic alterations for digestive NEC (*N* = 188). Upper panel shows the mutational burden per sample. Percentages on the right represent mutations frequency per gene. The panel under the oncoplot area is composed of two single row heatmaps showing celltype and primary tumour site.

The top-ten genetic most frequent alterations for NEC cases are shown in Fig. 1. Outcomes dependent on genetic alterations for NEC given cis/carboplatin and etoposide are summarised in Table 2. NEC with *TP53* mutations had a numerically lower RR than *TP53* wild-type cases (47 vs 31%, *p* = 0.108). We observed no responders among *BRAF* V600E mutated NEC (*n* = 9), compared to 41% among *BRAF* wild type (*n* = 101; *p* = 0.016) and double-wild type *BRAF/KRAS* had higher RR (45 vs 22%, *p* = 0.023) compared to NEC harbouring either mutation. No detrimental impact of BRAF status on OS was found. CNA did not significantly affect treatment outcome, but cases with *MYC* amplification had a numerically higher rate of immediate progression at first evaluation after initiation of chemotherapy (40 vs 23%, *p* = 0.099). For pancreatic NEC, neither *RB1* deletions nor *TP53* mutation affected response to cis/carboplatin and etoposide. We found a significantly higher rate of immediate progression in rectal NEC with *ARID1A* deletions (56 vs 33%, OR 6.43, 95%CI 1.05–39.33, *p* = 0.044) and a strong trend towards inferior RR (OR 0.19, 95%CI 0.03–1.02, *p* = 0.052).

**Table 2:** Treatment outcome after cis/carboplatin and etoposide for NEC according to genetic alterations (mutated/deleted/amplified vs wild type/not deleted/non-amplified cases) (*N* = 123).

	Response Rate	Immediate Progression	PFS (months)	OS (months)
*TP53* mut	31 vs 47%	39 vs 30%	3.7 vs 3.3	10.2 vs 7.7
*p*-value	0.108	0.317	0.238	0.068
*RB1* del	45 vs 39%	24 vs 37%	3.7 vs 3.5	9.4 vs 8.8
*p*-value	0.601	0.193	0.677	0.770
*ARID1A* del	36 vs 47%	31 vs 31%	5.0 vs 3.4	9.2 vs 9.4
*p*-value	0.289	0.976	0.233	0.800
*MYC* amp	33 vs 49%	40 vs 23%	2.7 vs 5.0	8.5 vs 10.2
*p*-value	0.136	0.099	0.289	0.142
*ATM* del	40 vs 43%	34 vs 29%	3.3 vs 4.1	10.4 vs 8.4.
*p*-value	0.786	0.569	0.994	0.788
*KDM5A* amp	40 vs 43%	31 vs 32%	3.7 vs 3.5	10.2 vs 8.4
*p*-value	0.808	0.926	0.752	0.756
*ESR1* del	40 vs 43%	29 vs 32%	3.4 vs 3.7	9.5 vs 8.9
*p*-value	0.808	0.678	0.991	0.956
*APC* mut	30 vs 40%	45 vs 31%	2.7 vs 3.5	9.3 vs 8.4
*p*-value	0.334	0.168	0.278	0.785
*KRAS* mut	25 vs 41%	48 vs 32%	2.8 vs 3.4	9.8 vs 8.3
*p*-value	0.160	0.126	0.341	0.926
*BRAF* mut	0 vs 41%	22 vs 37%	2.4 vs 3.4	8 vs 8.9
*p*-value	**0.016**	0.397	0.751	0.885
*KRAS/BRAF* mut	22 vs 45%	41 vs 32%	2.8 vs 3.7	9.8 vs 7.7
*p*-value	**0.023**	0.399	0.215	0.746

*PFS* progression free survival, *OS* overall survival.

Significant values are marked bold.

We found no significant correlation between genetic alterations and PFS as PFS was short regardless of any alteration. No significant correlation was found between genetic alterations and overall survival. There was a non-significant trend in univariate analyses towards longer survival for those harbouring *TP53* mutation 10.2 vs 7.7 m (*p* = 0.068). Tumour mutation burden (TMB) was generally low, somewhat higher for NEC (5.0) than for NET G3 (3.2). Comparing NEC cases above vs below mean TMB, we found no difference in RR, PFS or OS after cis/carboplatin and etoposide. 9/10 NEC cases with TMB ≥ 10 had MSI.

### Genetic alterations and treatment outcome according to NEC cell type

**Fig. 2:**
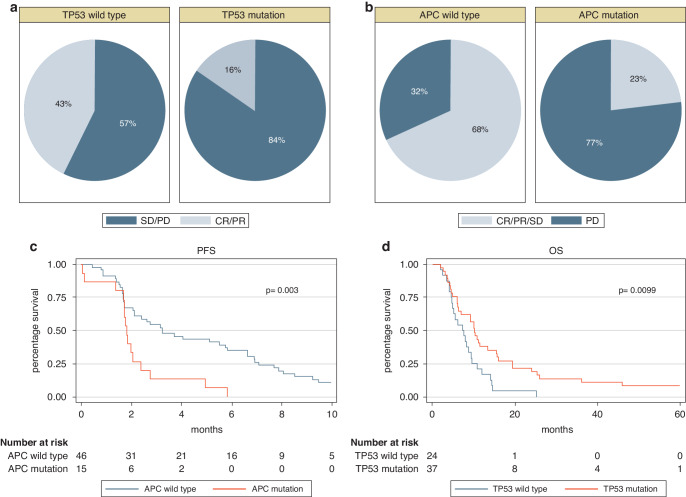
Efficacy to cis/carboplatin and etoposide and survival dependent on genetic alteration and NEC celltype. **a** Response to cis/carboplatin and etoposide according to *TP53* status for LC-NEC (*N* = 61). **b** Immediate progression to cis/carboplatin and etoposide according to *APC* status for LC-NEC (*N* = 61). **c** Progression free survival (PFS) after cis/carboplatin and etoposide for LC-NEC according to *APC* status (*N* = 61). **d** Overall survival (OS) for SC-NEC according to *TP53* status (*N* = 61).

For SC-NEC given cis/carboplatin and etoposide, *RB1* deletion predicted disease control (0% immediate progression vs 38%, *p* = 0.003) and *TP53* mutation was associated with longer OS (10.2 vs 7.3 m, *p* = 0.011). For LC-NEC, *TP53* mutated cases had lower RR (16 vs 43%, *p* = 0.033) whereas *APC* mutated cases had higher rate of immediate progression (77 vs 32%, *p* = 0.008) and shorter PFS (3.3 vs 1.8 m, *p* = 0.004). *ARID1A* deleted LC-NEC cases had longer PFS (5.0 vs 2.1 months, *p* = 0.032). Significant findings are illustrated in Fig. 2a–d, whereas Supplementary Table 1 summarise all outcomes for cis/carboplatin and etoposide for NEC dependent on cell type. In the MVA, *TP53* mutation and colon primary were significant predictors for inferior RR to cis/carboplatin and etoposide (*p* = 0.008 and 0.007 respectively; Table 3a). Only elevated LDH correlated with inferior survival (Table 3b).

**Table 3:** **a** Response rate (RR) to cis/carboplatin and etoposide for NEC (*N* = 123), univariate (partly shown in Table 2) and multivariate analyses. **b** Overall survival (OS) for advanced NEC given cis/carboplatin and etoposide (*N* = 123). Univariate analyses partly shown in Table 2. In multivariate analyses, only NEC with PS 0–1 is included (*N* = 90)

a			
		Univariate	Multivariate
	RR	OR, 95% CI, *p*-value	OR, 95% CI, *p*-value
*TP53* mutation vs wild type	31 vs 47%		**0.23, 0.08–0.69, 0.008**
*RB1* deletion vs non-deleted	45 vs 39%		1.11, 0.42–2.94, 0.835
*APC* mutation vs wild type	30 vs 40%		1.31, 0.40–4.29, 0.659
*MYC* amp vs non-amplified	33 vs 49%		0.81, 0.30–2.21, 0.682
Cell type, LC vs SC	26 vs 48%	**0.39, 0.17–0.86, 0.020**	0.43, 0.16–1.16, 0.096
Colon vs non-colonic	9 vs 44%	**0.13, 0.03–0.57, 0.007**	**0.08, 0.17–0.53, 0.007**

b			
		Univariate	Multivariate
	OS (months)	HR, 95% CI, *p*-value	HR, 95% CI, *p*-value
CRP (≥10 vs <10)	7.4 vs 11	**1.45, 1.01–2.11, 0.047**	1.33, 0.78–2.26, 0.301
LDH (elevated vs normal)	6.3 vs 11.1	**1.50, 1.04–2.18, 0.032**	**1.77, 1.05–2.99, 0.032**
PS (≥2 vs <2)	4.7 vs 10.2	**2.40, 1.57–3.67**,<**0.001**	
*TP53* mutation vs wild type	10.2 vs 7.7		0.81, 0.47–1.40, 0.457
*RB1* deletion vs non-deleted	9.4 vs 8.8		0.81, 0.47–1.38, 0.430
Cell type, LC vs SC	8.5 vs 9.2	1.12, 0.78–1.61, 0.535	1.44, 0.85–2.41, 0.172

Significant values are marked bold.

### NET G3

When assessing NET G3 for treatment outcome related to genetic alterations, we found no correlation to RR. *MEN1* mutation predicted immediate progression (*p* = 0.042), however only four cases (10%) harboured such a mutation. Shorter PFS was associated with *ARID1A* deletion (3.6 vs 12.6 m, *p* = 0.029), *ESR1* deletions (3.3. vs 9.8 m, *p* = 0.012) and *ATRX* mutation (2.6 vs 7.4 m, *p* = 0.022). After first-line chemotherapy, there was a trend towards longer survival for those without *ESR1* deletion (22.2 vs 8.3 m, *p* = 0.068). The most frequent genetic alterations for NET G3 are shown in Supplementary Fig. 1. Other factors affecting OS after chemotherapy were elevated LDH (8.3 vs 24.2 m, *p* = 0.008) and PS ≥ 2 (8.8 vs 24.2 m, *p* = 0.012). In a multivariate model with PS, LDH, Ki-67 (55% cut-off) and *ESR1* deletion, only elevated LDH (*p* = 0.047) kept its significant impact on OS whereas PS did not (*p* = 0.053). Among 13 NET G3 patients given cis/carboplatin and etoposide, the five with partial response had higher Ki-67 (mean 55.4 vs 35.9%, *p* = 0.082).

### *BRCA, ATM* and other potential targets in NET G3/NEC

Among our 229 digestive HG-NEN, 10 (5.3%) of NEC and 6 (14%) of NET G3 harboured *BRCA* 1/2 mutations. Among the 8 cases with available normal tissue, none were germline mutations. We found no association between *BRCA* 1/2 mutation and RR to first-line cis/carboplatin and etoposide, neither for NEC (33 vs 38%, *p* = 0.84) nor for the 16 NEN combined (44 vs 37%, *p* = 0.67). *BRCA* 1/2 mutations had no significant impact on PFS or OS for NEN or NEC. We found *ATM* mutations among 7/188 (4%) and *ATM* deletions among 66/151 (44%) of NEC. We found no association between *ATM* deletions or -mutations and RR, PFS or OS. We found other targetable mutations infrequently among NEC; *KRAS G12C* mutations in 3%, *RET* mutations in 3% and *HRAS* mutation in 0.5%, but no *ERBB2* amplification.

If adjustment for multiple testing, the only significant findings that withheld was longer survival for NET G3 compared to NEC and a significant longer survival for NEC with good performance status (PS 0–1).

## Discussion

Treatment of metastatic digestive NEC patients is a clinical challenge as many patients progress immediately at the first evaluation after initiating first-line chemotherapy. For those with an initial response, treatment resistance develops rapidly with median PFS only 4–5 months and median OS 11–12 months [8, 10, 11]. There is a high unmet need for better predictive markers, more efficient treatment and, in general, more knowledge on this disease. Few previous studies have assessed treatment outcome according to genetic alterations for digestive NEC. Our study present novel findings regarding outcome after cis/carboplatin and etoposide for digestive HG-NEN patients according to genetic alterations, and for NEC these findings seems partly dependent on cell-type.

*TP53 mutations* or abnormal p53 protein expression is associated with platinum resistance for several cancers [18, 19]. *TP53* mutations are frequent in NEC (51-89%) [15, 20–22]. In our study, *TP53* mutation predicted inferior RR to platinum/etoposide in multivariate analyses across all NEC but did not correlate to OS. *TP53* mutated SC-NEC, however, had a significantly better survival after platinum/etoposide. A recent study including pulmonary/digestive HG-NEN (*N* = 89) treated with cis/carboplatin and etoposide reported a numerical higher RR with inappropriate p53 (absence of or intense staining) [23]. A correlation between inferior survival and abnormal p53 expression/*TP53* mutations have previously been reported for digestive HG-NEN [22, 24]. Most but not all *TP53* mutations causes accumulation of p53 in the cell nuclei. This might lead to a relative discordance between protein p53 measurements and *TP53* mutation frequency, and mutational analyses has been recommended for evaluation of clinical outcomes [25]. The prognostic value of *TP53* mutations might depend on co-mutations and cancer type [26].

*BRAF V600E mutations* are frequent in colorectal NEC (28–47%), with a predominance in right-sided colon NEC [3, 27, 28]. *BRAF* mutations might be related to the particularly high frequency of treatment failure among colon NEC, where up to 65% have immediate progression on first-line treatment with cis/carboplatin and etoposide [6, 29]. In our study, no *BRAF* mutated NEC showed a response to first-line cis/carboplatin and etoposide, where 10/11 had colorectal origin. We found no OS differences dependent on *BRAF* status for NEC, a finding that could not be explained by possible differences in post first-line treatment. Our finding is in huge contrast to the substantial shorter survival found when *BRAF* mutation is present in metastatic colorectal adenocarcinoma supporting that colorectal NEC and adenocarcinoma are separate entities. Case studies have reported benefit of BRAF/MEK inhibition in *BRAF* mutated colorectal NEC [27, 30], and a BRAF/EGFR-inhibitor combination is approved for *BRAF* mutated CRC, not limited to adenocarcinomas. FDA recently approved a BRAF/MEK-inhibitor combination for *BRAF* mutated tumours, regardless of primary tumour site. Given our findings, *BRAF* V600E inhibition may be an attractive strategy for future trials on NEC.

*RB1 deficiency* is proposed as a marker predicting platinum effect for NEC, but results are conflicting [16, 31, 32]. *RB1* mutations are infrequent in digestive NEC (14-25%), but *RB1* deficiency, assessed by both genetic alterations and protein expression is more commonly reported (36-86%). The frequency of *RB1* inactivation seems to differ according to primary site and is higher in SC compared to LC-NEC [3, 15, 33, 34]. In our data, no SC-NEC harbouring *RB1* deletion experienced immediate progression on first-line cis/carboplatin and etoposide, as opposed to 38% of those without deletions. This is in line with previous published data on SCLC where *RB1* mutations have been associated with improved responses, PFS and OS with platinum/etoposide [35]. We failed, however, to reproduce the impact on PFS and OS. While there are several mechanisms causing lack of proper pRb function, our data included only deletions and mutations and we may therefore underestimate the rate of pRb inactivation detected by immunohistochemical analysis. In a report on pancreatic NEC given platinum-treatment, RR was significantly higher for *RB1* loss and/or *KRAS* mutation. Both *RB1* loss and *KRAS* mutation predicted poor prognosis in univariate analyses, whereas only *RB1* loss was prognostic in a multivariate model [16]. In a study on 54 HG-NEN,*TP53* and/or *BRAF* mutations and immunohistochemical loss of Rb1- or p53 predicted shorter survival in univariate analyses [21].

*MYC amplification* is commonly described in both lung- and extra-pulmonary NEC, and proposed as a possible future therapy target [36]. Altered MYC has been linked to treatment resistance in several types of cancer [37]. A phase I study on MYC inhibition (OMO-103) reported effect among 22 patients with advanced solid tumours [38]. In our study, 7 (24%) of NET G3 and 74 (49%) of NEC had *MYC* amplification. Besides a non-significant trend towards decreased efficacy of cis/carboplatin and etoposide for *MYC* amplified NEC, we found no association between *MYC* status and treatment outcome nor survival.

*BRCA mutations and ATM alterations*. *BRCA* mutations are extremely rare in digestive adenocarcinomas (0.5-2%) [39], but its incidence among digestive HG-NEN has not been reported prior. Our findings of *BRCA* 1/2 mutations in 5.3% of NEC and 14.6% of NET G3 seem higher than for adenocarcinoma. *ATM* is mutated in ~5% of all cancers, but up to 10% of digestive adenocarcinoma [40]. Among 227 metastatic colorectal cancer, ATM mutations (15%) were correlated with superior OS [41]. We found *ATM* mutations in 4% and *ATM* deletions among 44% of our NEC cases. *BRCA1/2* and *ATM* might be future new treatment targets in digestive NEC. *BRCA* 1/2 genes are critical in repair of double strand breaks through the homologous recombination repair (HRR) pathway. Alterations in these genes serve as predictive biomarkers to both platinum and PARP inhibitors [42]. Whether PARP inhibition has a role for treatment of NEC is, to our knowledge, not yet explored. *ATM* is also involved in HRR; however, its role as a predictive biomarker to DNA damage response agents is debated [43]. For both *BRCA1/2* mutations and *ATM* alterations, we found no correlation to neither RR, PFS nor survival in platinum/etoposide treated cases. ATR protein inhibitors are under phase I/II investigation in NEC [44]. In a phase I study of ATR inhibitor elimusertib in solid tumours, all obtaining a partial response harboured *ATM* mutation or *ATM* protein loss [45].

### NEC cell type

The clinical significance of digestive NEC cell-type is still unknown and both cell types are at present treated similarly. Some prior studies have reported a lower RR to platinum/etoposide in LC-NEC and an association with longer OS compared to SC-NEC [4, 46]. For SC-NEC we found a significantly higher RR compared to LC-NEC, a trend towards longer PFS but no impact on OS. When assessing genetic alterations according to cell-type for NEC, we found several correlations to outcome after cis/carboplatin and etoposide. For SC-NEC, *RB1* deletion predicted disease control, *ARID1A* deletion immediate progression and *TP53* mutation a significant better survival after platinum/etoposide. For LC-NEC, *APC* mutations predicted immediate progression and shorter PFS, whereas *ARID1A* deletion predicted disease control and longer PFS. Our results support that the classification of digestive NEC into large- and small cell is molecularly and clinically relevant. To our knowledge no prior study has reported on cell-specific outcome to treatment dependent on genetic alterations for NEC, and our findings should be validated in future trials.

### NET G3

Surprisingly, NET G3 had a similar RR to platinum/etoposide compared to NEC, probably due to a high Ki-67 among NET G3 responders. This finding supports the updated NCCN guidelines suggesting consideration of platinum-compounds for NET G3 with high Ki-67 or aggressive behaviour [47]. For NET G3, we found no correlations between genetic alterations and response to chemotherapy or OS. *ATRX* mutation, *ARID1A*- and *ERS1* deletions were associated with shorter PFS, but the sample size in these subgroups were very small. There are few published data on genomic aberrations and treatment outcomes for NET G3. In a recently presented study, a genomic signature of *MEN1* mutation and *DAXX*-wild type correlated with longer PFS for pancreatic NET after capecitabine/ temozolomide treatment [48].

### Clinical biomarkers

Poor PS is a well-known prognostic factor for digestive HG-NEN [6, 49]. In our data, LDH, CRP and PS were prognostic for NEC given cis/carboplatin and etoposide. In a multivariate model including only PS < 2, only elevated LDH was significantly associated with inferior survival. Elevated LDH has previously been shown to be prognostic for digestive NEC, and is one of the five variables included in the GI-NEC score [50]. When searching for novel prognostic molecular markers, still including the known traditional clinical markers seems important.

### Strengths and limitations

We present prospectively collected data on one of the largest digestive HG-NEN cohorts described to date. However, sample size in several subgroups is still small. Our patient characteristics are similar to other studies published, indicating that the study cohort represents a real-life occurrence of digestive HG-NEN. Pathological expertise reassessed all cases according to the 2019 WHO classification. Presented genetic aberrations were limited to CNA and mutations, and a multi-omics approach would have provided a more in-depth information on aberrations that could influence treatment outcomes. Information on Rb-protein expression by immunohistochemistry could have added additional information but was not available due to the multi-centric approach. Multiple testing might increase the risk of false positive results. Although this is not normally done, we explored for this using the Benjamini- Hochberg procedure. Most of our results lost its significance after such an adjustment, likely due to our many small subgroups.

## Summary

Correlations between genetic alterations and response/immediate progression to first-line cis/carboplatin and etoposide were frequent, but rarely affected PFS. In MVA, *TP53* mutation was a significant predictor for inferior RR to cis/carboplatin for NEC. Except for a longer survival for *TP53* mutated SC-NEC after platinum/etoposide, none of the investigated genetic alterations in our study was associated with a significant impact on OS in NEC. When separating NEC according to cell-type, several genetic alterations were correlated to efficacy indicating that the classification of digestive NEC into large cell and small cell is molecularly and clinically relevant. *ATM* alterations and *BRCA* mutations could be potential targets for novel therapeutic approaches. Several NET G3 genetic alterations were associated with PFS, however NET G3 cases were limited. When searching for novel prognostic molecular markers, still including the known traditional clinical markers seems important. The reason for the lack of substantial correlations between genetic alterations and OS is not obvious but could be due to the extreme aggressiveness of the disease with a very short PFS and OS. In future search for markers predicting treatment success, a multi-omics approach might be a way to better uncover the molecular mechanism behind the poor treatment outcome for digestive NEC.

### Supplementary information

Supplementary Fig 1 legend

Supplementary Table 1 Treatment outcome to ciscarboplatin and etoposide according to genetic alteration stratified by NEC cell type

Supplementary Fig 1

## Data Availability

The data generated and analysed in this study are available from the corresponding author upon reasonable request.
