# Supplementary material for: Treatment outcome according to genetic tumour alterations and clinical characteristics in digestive high-grade neuroendocrine neoplasms
Source: Br J Cancer. 2024 Jun 22;131(4):676–84. doi: 10.1038/s41416-024-02773-w (PMC11333587; doi:10.1038/s41416-024-02773-w)
Supplement: Supplementary file 1 — Supplementary Fig 1 legend [file 41416_2024_2773_MOESM1_ESM.docx]

Legends supplementary figure

Fig 1. Oncoplot showing the top-ten alterations among 41 digestive NET G3
