# Supplementary material for: Treatment outcome according to genetic tumour alterations and clinical characteristics in digestive high-grade neuroendocrine neoplasms
Source: Br J Cancer. 2024 Jun 22;131(4):676–84. doi: 10.1038/s41416-024-02773-w (PMC11333587; doi:10.1038/s41416-024-02773-w)
Supplement: Supplementary file 2 — Supplementary Table 1 Treatment outcome to ciscarboplatin and etoposide according to genetic alteration stratified by NEC cell type [file 41416_2024_2773_MOESM2_ESM.docx]

Supplementary Table 1.

Treatment outcome to cis/carboplatin and etoposide according to genetic alteration stratified by NEC cell type (N= 122). All findings listed as OR/ HR, 95% CI, p-value.

**a) LC-NEC (N=61)**

|  | **TP53 mutation** | **APC mutation** | **KRAS mutation** | **BRAF mutation** | **RB1 deletion** |
| --- | --- | --- | --- | --- | --- |
| Response | **0.25 (0.07- 0.89, 0.033)** | 0.20 (0.023- 1.68, 0.137) | 0.48 (0.09- 2.54, 0.391) | no responses | 0.45 (0.13- 1.61, 0.221) |
| Progression | 2.14 (0.69- 1.93, 0.188) | **7.18 (1.69- 30.55, 0.008)** | 1.83 (0.52- 6.41, 0.350) | 0.49 (0.09- 2.82, 0.428) | 1.64 (0.50- 5.38, 0.417) |
| PFS | 1.14 (0.67- 1.93, 0.627) | **2.58 (1.36- 4.93, 0.004)** | 1.14 (0.62- 2.07, 0.680) | 0.85 (0.40- 1.80, 0.676) | 1.15 (0.66- 2.00, 0.621) |
| OS | 0.99 (0.59- 1.69, 0.911) | 1.37 (0.75- 2.50, 0.310) | 1.09 (0.59- 2.00, 0.078) | 0.84 (0.39- 1.79, 0.658) | 1.29 (0.74- 2.26, 0.364) |
|  | **MYC amplification** | **ARID1A deletion** | **ESR1 deletion** | **KDM5A amplification** | **ATM deletion** |
| Response | 0.45 (0.13- 1.61, 0.221) | 1.04 (0.29- 3.69, 0.955) | 0.75 (0.21- 2.66, 0.656) | 0.71 (0.20- 2.61, 0.611) | 0.75 (0.21- 2.66, 0.656) |
| Progression | 1.87 ( 0.55- 6.33, 0.316) | 0.33 (0.10- 1.13, 0.078) | 0.59 (0.18- 1.96, 0.393) | 2.38 (0.71- 7.92, 0.159) | 2.23 (0.67- 7.40, 0.192) |
| PFS | 0.88 (0.50- 1.53, 0.644) | **0.53 (0.30- 0.95, 0.032)** | 0.68 (0.39- 1.19, 0.174) | 0.92 (0.53- 1.61, 0.774) | 1.11 (0.64- 1.92, 0.723) |
| OS | 1.18 (0.67- 2.07, 0.560) | 0.79 (0.46- 1.37, 0.411) | 0.70 (0.40- 1.22, 0.205) | 0.90 (0.51- 1.57, 0.700) | 1.12 (0.64- 1.97, 0.695) |
|  |  |  |  |  |  |

**b) SC-NEC (N=61)**

|  | **TP53 mutation** | **APC mutation** | **KRAS mutation** | **BRAF mutation** | **RB1 deletion** |
| --- | --- | --- | --- | --- | --- |
| Response | 0.89 (0.30- 2.60, 0.830) | 0.8 (0.26- 2.47, 0.698) | 0.46 (0.12- 1.74, 0.251) | no responses | 2.36 (0.67- 8.39, 0.183) |
| Progression | 0.96 (0.29- 3.22, 0.947) | 1.2 (0.37- 3.92, 0.853) | 2.43 (0.63- 9.34, 0.197) | no progressions | no progressions |
| PFS | 0.61 (0.35- 1.04, 0.071) | 0.88 ( 0.51- 1.54, 0.664) | 1.28 (0.69- 2.40, 0.425) | 3.6 (0.83- 15.4, 0.086) | 0.73 (0.40- 1.35, 0.315) |
| OS | **0.49 (0.28- 0.85, 0.011)** | 0.87 (0.50- 1.53, 0.637) | 0.92 (0.49- 1.71, 0.792) | 3.30 (0.78- 13.9, 0.104) | 0.84 (0.46- 1.55, 0.575) |
|  | **MYC amplification** | **ARID1A deletion** | **ESR1 deletion** | **KDM5A amplification** | **ATM deletion** |
| Response | 0.91 (0.26- 3.25, 0.890) | 0.49 (0.14- 1.72, 0.264) | 2.00 (0.49- 8.09, 0.331) | 1.10 (0.31- 3.86, 0.879) | 1.06 (0.31- 3.61, 0.929) |
| Progression | 1.6 (0.36- 7.18, 0.539) | 4.6 (0.96- 22.16, 0.057) | 0.66 (0.12- 3.74, 0.636) | 0.15 (0.02- 1.34, 0.089) | 0.60 (0.13-2.82, 0.517) |
| PFS | 1.6 (0.89- 3.04, 0.116) | 0.95 (0.51- 1.78, 0.879) | 1.48 (0.76- 2.85, 0.244) | 0.96 (0.51- 1.79, 0.891) | 0.80 (0.43- 1.49, 0.488) |
| OS | 1.57 (0.86- 2.89, 0.144) | 1.10 (0.59- 2.05, 0.763) | 1.56 (0.82- 2.99, 0.175) | 0.94 (0.51- 1.73, 0.837) | 0.76 (0.41- 1.43, 0.402) |
|  |  |  |  |  |  |
