# Supplementary figures and images for: Treatment outcome according to genetic tumour alterations and clinical characteristics in digestive high-grade neuroendocrine neoplasms
Source: Br J Cancer. 2024 Jun 22;131(4):676–84. doi: 10.1038/s41416-024-02773-w (PMC11333587; doi:10.1038/s41416-024-02773-w)

Altered in 32 (78.05%) of 41 samples.

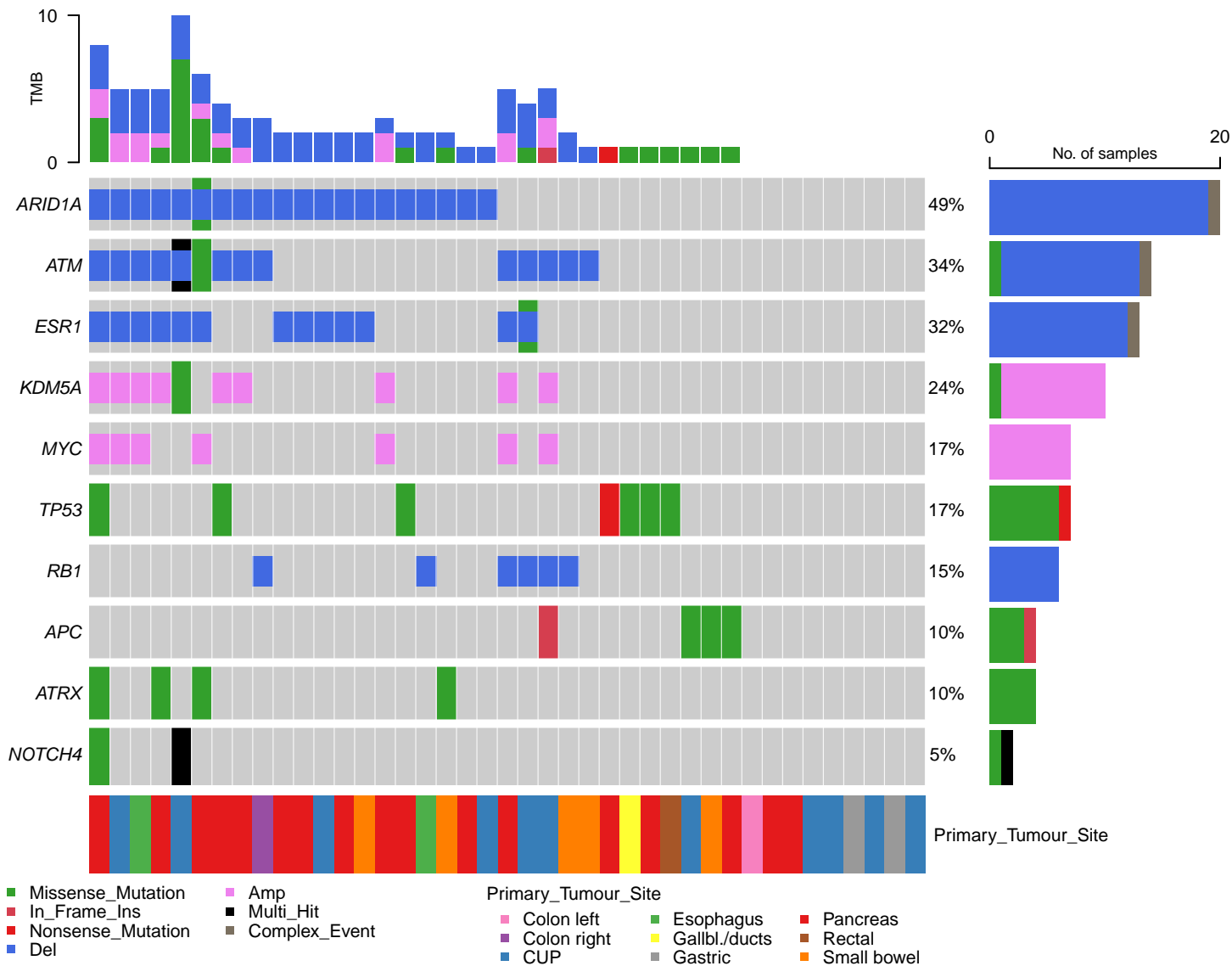

Supplement: Supplementary file 3 — Supplementary Fig 1 [file 41416_2024_2773_MOESM3_ESM.pdf]
